# Long term headache duration is a factor predicting nonresponse to detoxification and advice in medication overuse headache

**DOI:** 10.1186/1129-2377-15-88

**Published:** 2014-12-15

**Authors:** Stefano Caproni, Elisa Bianchi, Letizia M Cupini, Ilenia Corbelli, Ettore Beghi, Paolo Calabresi, Paola Sarchielli, SAMOHA Study Group

**Affiliations:** 1 Clinica Neurologica, Azienda Ospedaliera, Università di Perugia, Sant’Andrea delle Fratte, 06156 Perugia, Italy; 2 IRCCS - Istituto di Ricerche Farmacologiche “Mario Negri”, Via La Masa, 19-20156 Milano Milano, Italy; 3 Centro Cefalee e Malattie Cerebrovascolari, Ospedale S. Eugenio, Piazzale dell’Umanesimo, 10 00144 Roma, Italy; 4 IRCCS Fondazione “S. Lucia”, Via Ardeatina, 306, 00142 Roma, Italy

**Keywords:** MOH, Detoxification, Advice, Headache duration, Treatment

## Abstract

**Background:**

Medication overuse headache (MOH) is a very heterogeneous disorder for which a recommended treatment is not yet available. The purpose of this study was to investigate any possible roles of demographic and clinical characteristics of MOH patients that might predict a response to detoxification and advice with or without preventive treatment.

**Findings:**

This ancillary study is part of the Sodium vAlproate in the treatment of Medication Overuse HeadAche (SAMOHA) study that randomized 88 MOH patients for 3-month treatment period with sodium valproate (VPA) (800 mg/day) or placebo after a 6-day outpatient detoxification regimen. Demographic and clinical characteristics obtained on patients from both study arms were analyzed to point out an association with the response to the treatment. While for patients from VPA arm no significant results were obtained, comparing responders to non-responders to detoxification and advice to withdraw from MOH, a significant difference in headache duration was observed. Specifically, the efficacy of such treatment resulted ineffective in headache lasting longer than 30 years.

**Conclusions:**

Our findings suggest that the benefit from detoxification and advice can be excluded in MOH of long duration. Therefore, a preventive treatment is suggested particularly for these patients.

## Background

Medication-overuse headache (MOH) is a secondary chronic headache having a relevant impact in clinical practice, with a prevalence of 1-2% in the general population [1–3]. MOH is a very heterogeneous disorder for which a recommended treatment is not yet available. The management of MOH currently represents a difficult challenge for clinicians and headache experts [4]. A possible treatment choice is detoxification and the initiation of a prophylactic therapy [5, 6]. Nevertheless, the timing of this prophylactic treatment after detoxification remains one of the most debated aspects of MOH care [7] and a matter of concern among patients [8]. In a recent randomized open-label trial, a structured inpatient detoxification program, characterized by the immediate start of a preventive treatment, resulted more effective than advice alone or a structured outpatient program to achieve drug withdrawal [9]. Likewise, the Sodium vAlproate in the treatment of Medication Overuse HeadAche (SAMOHA) study recently showed the efficacy and safety of sodium valproate (VPA), compared to placebo, after detoxification in the short-term treatment of MOH patients with a history of migraine without aura [10]. In light of this, we analyzed data obtained from patients in both arms of the SAMOHA study, in search of demographic and clinical characteristics which could predict a response to detoxification and advice to withdraw from drug abuse.

## Methods

### Standard protocol approvals, registrations, and patient consents

As an ancillary study, the protocol was submitted for approval to the Ethics Committees of each participating center. The SAMOHA trial was registered on the European Union Drug Regulating Authorities Clinical Trials website (EudraCT code 2007-006773-92; https://www.clinicaltrialsregister.eu/ctr-search/trial/2007-006773-92/IT). The SAMOHA study was conducted in accordance with the Declaration of Helsinki and its amendments (Seoul, October 2008). All patients provided their written consent to participate in the study.

### Subjects

This is an ancillary study related to the SAMOHA study, a multicenter, randomized, double-blind, placebo-controlled trial. After a 4-week baseline period (during which no study medication was given) 88 MOH patients received a 6-day outpatient detoxification regimen and then a 3-month treatment period using VPA (800 mg/day), followed by a 3-month follow-up (to verify the possibility of a carry-over effect of treatment). After the detoxification phase, patients were advised to discontinue the overused medication. Although acute medications were consented, no specific symptomatic drugs were recommended during follow-up.

The 3-month responder rate (the proportion of patients achieving ≥50% reduction in the number of days with headache per month) was 23.8% for the placebo arm and 45% for the VPA arm, while after the 3-month follow-up period this rate did not differ between the two groups [10].

### Statistical analysis

Demographic (sex, age), clinical (body mass index, comorbidity, surgery) and headache characteristics (frequency, intensity, total and MOH duration, overused drugs) in Responders and Non-Responders (R and NR, respectively) from both study arms were compared. Psychiatric comorbidities and personality traits were assessed using the following inventories: Modified MINI-International Neuropsychiatric Interview [11]: 0 vs. 1+ psychiatric disorders; Beck Anxiety Inventory [12]: 0-21vs. 22+; Beck Depression Inventory [13]: 0–10, 11–20, 21+, Yale Brown Obsessive-Compulsive Scale [14]: 0 vs. 1+; Leeds Dependence Questionnaire [15]: <10, 10–22, >22. All variables were categorized and assessed using the Fisher exact test. Statistical significance was set at the 5% level. Data was analyzed using the SAS package for PC (SAS Institute, Cary, NV; version 9.2).

## Findings

For patients in the VPA arm, no differences between R and NR were observed fordemographic, clinical and headache characteristics (Table 1). Conversely, for patients in the placebo group, treated by detoxification and advice to withdraw from MOH, a significant difference between R and NR was found only for headache duration, while all the other variables were evenly distributed (Table 2). Specifically, in MOH patients with a history of migraine longer than 30 years, the probability of response to detoxification and advice to withdraw from drug abuse was irrelevant.

**Table 1:** **Comparison of demographic and clinical characteristics between responders and non-responders from VPA arm**

	Responders		Non-responders		
	(N = 18)		(N = 26)		
	N	%	N	%	p-value
Sex					
F	14	77.8	21	80.8	0.8089
M	4	22.2	5	19.2	
Age class (years)					
18-34	2	11.1	3	11.5	0.8259
35-44	9	50	11	42.3	
45-54	4	22.2	9	34.6	
55-64	3	16.7	3	11.5	
BMI class					
(<18) underweight	0	0	1	3.9	0.4251
(18–24.9) normal weight	8	44.4	16	61.5	
(25–29.9) overweight	8	44.4	6	23.1	
(>30) obese	2	11.2	3	11.5	
Comorbidity					
No	17	94.4	20	76.9	0.1182
Yes	1	5.6	6	23.1	
Surgery					
No	2	11.8	6	24	0.3216
Yes	15	88.2	19	76	
Missing	1		1		
Headache days/months (V1)					
15-25	12	66.7	14	53.8	0.4405
>25	6	33.3	12	46.2	
Headache intensity (V1)					
Slight	0	0	1	3.8	0.6987
Moderate	6	33.3	8	30.8	
Severe	12	66.7	17	65.4	
Headache days/month (V2)					
15-25	17	94.4	19	73.1	0.1216
>25	1	5.6	7	26.9	
Headache intensity (V2)					
Slight	3	16.7	3	11.5	0.7040
Moderate	11	61.1	19	73.1	
Severe	4	22.1	4	15.4	
Headache duration (years)					
0-10	1	5.6	5	19.2	0.4249
11-20	6	33.3	7	26.9	
21-30	7	38.9	6	23.1	
>30	4	22.2	8	30.7	
MOH duration (years)					
<1	2	11.1	4	15.4	0.5182
1-3	4	22.2	6	23.1	
3-5	7	38.9	5	19.2	
>5	5	27.8	11	42.3	
Overused drugs					
Analgesics >14d	9	50	6	23.1	0.2703
Analgesics combinations >9d	2	11.1	3	11.5	
Drug combinations >9d	2	11.1	7	26.9	
Triptan combinations >9d	5	27.8	10	38.5	

VPA: sodium valproate.

BMI: body mass index.

V1: baseline first visit.

V2: second visit at the start of detoxification.

d: days.

**Table 2:** **Comparison of demographic and clinical characteristics between responders and non-responders from placebo arm**

	Responders		Non-responders		
	(N = 10)		(N = 34)		
	N	%	N	%	p-value
Sex					
F	10	100	24	70.6	0.0853
M	0	0	10	29.4	
Age class (years)					
18-34	4	40	4	11.8	0.2563
35-44	2	20	12	35.3	
45-54	3	30	14	41.2	
55-64	1	10	4	11.7	
BMI class					
(<18) underweight	0	0	2	6.1	0.9166
(18–24.9) normal weight	6	60	21	63.6	
(25–29.9) overweight	3	30	6	18.2	
(>30) obese	1	10	4	12.1	
NS			1		
Comorbidity					
No	6	60	23	67.7	0.7138
Yes	4	40	11	32.3	
Surgery					
No	2	22.2	15	44.1	0.2807
Yes	7	77.8	19	55.9	
Missing	1				
Headache days/months (V1)					
15-25	6	60	20	58.8	1.000
>25	4	40	14	41.2	
Headache intensity (V1)					
Slight	1	10	1	2.9	0.1818
Moderate	2	20	16	47.1	
Severe	7	70	17	50	
Headache days/month (V2)					
<15	0	0	2	5.9	1.000
15-25	6	60	20	58.8	
>25	4	40	12	35.3	
Headache intensity (V2)					
Slight	0	0	3	8.8	0.2947
Moderate	8	80	17	50	
Severe	2	20	14	41.2	
Headache duration (years)					
0-10	2	20	5	14.7	0.0229
11-20	1	10	6	17.6	
21-30	7	70	9	26.5	
>30	0	0	14	41.2	
MOH duration (years)					
<1	1	10	5	14.7	0.9159
1-3	4	40	11	32.4	
3-5	1	10	7	20.6	
>5	4	40	11	32.3	
Overused drugs					
Analgesics >14d	3	30	12	35.3	0.7442
Analgesics combinations >9d	2	20	5	14.7	
Drug combinations >9d	4	40	9	26.5	
Triptan combinations >9d	1	10	8	23.5	

BMI: body mass index.

V1: baseline first visit.

V2: second visit at the start of detoxification.

d: days.

With reference to psychiatric comorbidity and personality traits, none of the assessed inventories differed between R and NR (data not shown).

## Discussion

To our knowledge, this is one of the few studies analyzing a possible correlation between demographic, clinical and headache characteristics of MOH patients and response to treatment. Our findings suggest that, in presence of a headache lasting more than 30 years, patients seem to have no benefit from detoxification and advice to withdraw from drug abuse, despite the possible placebo effect. Previously, in a cross-sectional epidemiological survey, the severity of dependence scale score could predict successful prognosis related to detoxification in MOH patients without other complicating secondary headaches [16].

The challenge of achieving a stable withdrawal from medication overuse is one of the most disputed aspects in the treatment of MOH. This lack of agreement is, for the most part, due to the heterogeneity of patients, overused medications, detoxification procedures and protocol designs [17].

Detoxification is often the first step in withdrawal from medication overuse. In this regard, some studies have suggested benefits from detoxification followed by advice for MOH [9]. Other studies have supported the efficacy of simple advice as a withdrawal strategy in MOH [18, 19]. However, in most of these studies there were no controls nor comparisons between responders and non-responders.

The results from our study suggest that in MOH patients with a history of headache longer than 30 years, even in the absence of relevant comorbidities, there is no benefit from detoxification and advice without preventive therapy. This lack of benefit could be due to the lingering central sensitization underlying the pathophysiology of MOH [17]. In these cases, we would suggest an immediate initiation of a prophylactic treatment, to avoid a relapse of drug abuse. This therapeutic approach could lead to a swifter clinical response, better quality of life and economic savings. It is also interesting that MOH duration did not differ in R and NR; this aspect should be further investigated, in order to hypothesize common or different pathophysiological mechanisms for migraine and MOH.

Our study has some limitations. First, the available sample size is incompatible with definitive results regarding the demographic and clinical characteristics we investigated. Given the small numbers, we could not perform a multivariate analysis of our data. So, headache duration may be masked by other confounding variables. Second, some of the patients in our study were included in the placebo arm of the SAMOHA study and, as such, they did not know if they were untreated. Even if a placebo effect cannot be ignored in MOH patients, the validity of our research hypothesis is weakened but not entirely excluded. Third, as our study analyzed the short-term efficacy of treatment, the long-term response to detoxification and advise is unknown.

In conclusion, in the case of headache of long duration our findings suggest that the benefit from detoxification and advice can be excluded in MOH patients. Therefore, a preventive treatment is suggested for these patients. Larger studies with longer follow-up are needed on this issue as their results could lead to a better management of MOH patients.

## Abbreviations

MOH: Medication-overuse headache
SAMOHA: Sodium vAlproate in the treatment of Medication Overuse HeadAche
VPA: Sodium valproate
R: Responders
NR: Non-responders.
